# The Different Function of Single Phosphorylation Sites of *Drosophila melanogaster* Lamin Dm and Lamin C

**DOI:** 10.1371/journal.pone.0032649

**Published:** 2012-02-29

**Authors:** Magdalena Zaremba-Czogalla, Katarzyna Piekarowicz, Katarzyna Wachowicz, Katarzyna Kozioł, Magda Dubińska-Magiera, Ryszard Rzepecki

**Affiliations:** Laboratory of Nuclear Proteins, Faculty of Biotechnology, University of Wroclaw, Wroclaw, Poland; University of Colorado, Boulder, United States of America

## Abstract

Lamins' functions are regulated by phosphorylation at specific sites but our understanding of the role of such modifications is practically limited to the function of cdc 2 (cdk1) kinase sites in depolymerization of the nuclear lamina during mitosis. In our study we used *Drosophila* lamin Dm (B-type) to examine the function of particular phosphorylation sites using pseudophosphorylated mutants mimicking single phosphorylation at experimentally confirmed *in vivo* phosphosites (S^25^E, S^45^E, T^435^E, S^595^E). We also analyzed lamin C (A-type) and its mutant S^37^E representing the N-terminal cdc2 (mitotic) site as well as lamin Dm R^64^H mutant as a control, non-polymerizing lamin. In the polymerization assay we could observe different effects of N-terminal cdc2 site pseudophosphorylation on A- and B-type lamins: lamin Dm S^45^E mutant was insoluble, in contrast to lamin C S^37^E. Lamin Dm T^435^E (C-terminal cdc2 site) and R^64^H were soluble *in vitro*. We also confirmed that none of the single phosphorylation site modifications affected the chromatin binding of lamin Dm, in contrast to the lamin C N-terminal cdc2 site. *In vivo*, all lamin Dm mutants were incorporated efficiently into the nuclear lamina in transfected *Drosophila* S2 and HeLa cells, although significant amounts of S^45^E and T^435^E were also located in cytoplasm. When farnesylation incompetent mutants were expressed in HeLa cells, lamin Dm T^435^E was cytoplasmic and showed higher mobility in FRAP assay.

## Introduction

**Figure 1:**
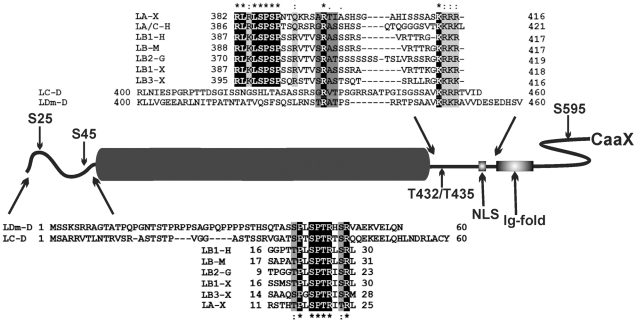
Comparison of the conserved amino acid sequences located in the N-terminal and C-terminal fragment of lamins containing the cdc2 kinase site, using Clustal W. A schematic view of the lamin monomer with *in vivo* identified embryonic phosphorylation sites as well as NLS, Ig-fold and CaaX motif are shown. Identical amino acids are marked by black boxes, similar by shadowed boxes. Four conserved regions were identified in vertebrate lamins and three in *Drosophila* lamins. The first region in all lamins also contains the N-terminal cdc2 site (SPTR motif). The second region located at the very beginning of the tail domain is present in vertebrates only and contains their C-terminal cdc2 site (SPXXR motif). The *Drosophila* C-terminal cdc2 site is located partially in the third conservative region (T/SRAT/S sequences) – TPSR motif for lamin Dm and TPSGR motif in lamin C. There is also an alternative C-terminal cdc2 site in lamin C (S^405^ in SPGR motif ). LDm-D *Drosophila melanogaster* lamin Dm, LC-D lamin C from fruit fly, LA/C-H human lamin A/C, LB1-H human lamin B1, LB-M mouse lamin B, LB2-G chicken lamin B2, LB1-X *Xenopus* lamin B1, LB3-X *Xenopus* lamin B3, LA-X *Xenopus* lamin A.

Lamins are the major components of the nuclear lamina, a dense, filamentous meshwork which provides structural support for the nuclear envelope (NE), although a fraction of lamins are present in the nuclear interior as well. Lamins serve as an organizing center for essential cellular processes such as transcription, DNA replication, cell differentiation, nuclear migration and others [1]–[4]. Mutations in nuclear lamina genes may cause a wide range of heritable human diseases generally termed laminopathies [5]. Lamins belong to the type V intermediate filaments. They contain a central α-helical rod domain flanked by a short N-terminal head domain and a carboxy-terminal tail domain, with NLS (nuclear localization signal or sequence) signal, conserved immunoglobulin fold and CaaX box (Figure 1) [6], [7]. The central rod domain, which is necessary for the coiled-coil dimer formation, comprises four coiled-coil domains separated by flexible linker regions. Lamins can also associate longitudinally into head-to-tail polymers. In these interactions, binding sites located on the ends of the rod domain as well as in part of the head and tail domains near to the central domain are involved [8]–[13].

The level and pattern of phosphorylation of lamins vary throughout the cell cycle [14]. Phosphorylation of amino acid residues in the head and in the tail domain, located next to the central coiled-coil domain, inhibit lamin polymerization and might disrupt the nuclear lamina network during mitosis [11], [15]–[20]. Mammalian mutant lamin A with removed cdc2 kinase (cell division control protein 2 homolog, also known as cyclin dependent kinase 1) phosphorylation sites caused aberrant mitosis when injected into mammalian cultured cells [21]. This was probably because of the lack of ability of lamins to depolymerize, but neither the mechanism nor any evidence has been revealed [17], [20]. It is known that during mitosis the nuclear envelope structure is affected by mitotic microtubule-induced tearing of the nuclear lamina [22]–[23], although this must be accompanied by lamin phosphorylation on mitotic site(s) in order to weaken the strength of lamin polymers. Interestingly, it is possible that, for nuclear lamina depolymerization, not only proper (mitotic) sites for a particular lamin must be phosphorylated but also the interphase pattern of phosphorylation must be removed [24]. As well, at the end of mitosis, mitotic sites are dephosphorylated, and the nuclear envelope assembles again to encircle the decondensing chromatin. Some evidence has suggested that type 1 protein phosphatase (PP1) is the major mitotic lamin phosphatase responsible for removal of mitotic phosphates [25]–[26].

While the function of mitotic lamin phosphorylation is well characterized, very little is known about the possible role of specific lamin modification during interphase. One of them is the control of subcellular distribution of lamins by regulation of their uptake into the nucleus [27], [28]. The role of lamin hyperphosphorylation during apoptosis [29]–[30] and virus infection [31]–[33] has also been investigated. It was also suggested that phosphorylation could regulate lamin binding to chromatin [34]. In mouse myoblast cell line C2C12 lamin A is phosphorylated at S^404^ by Akt kinase in response to insulin [35]. Phosphorylation of human lamin A (S^392^) and B1 (S^393^) by cdk5 kinase can directly cause neuronal cell death observed in neurodegenerative diseases, including Alzheimer's disease [36]. Unfortunately, so far almost nothing is known about the role of lamin phosphorylation in *Drosophila melanogaster*.

Fruit fly has two lamin genes coding for lamin Dm and lamin C, which are equivalents of vertebrate B- and A-type lamins, respectively. There are at least three posttranslationally modified lamin Dm isoforms: Dm_1_, Dm_2_ and Dm_mit_ [37]–[38]. Lamins Dm_1_ and Dm_2_ occur as an insoluble polymer that is mostly underlying the nuclear envelope during interphase [38]. Dm_2_ isoform arises from Dm_1_ by phosphorylation on the N-terminal site at or around S^25^ [38]. Both aforementioned isoforms, contrary to lamin C, interact with nucleic acids *in vivo*. During mitosis this interaction is lost [39]. Lamins Dm_1_ and Dm_2_ change during mitosis into a soluble form (lamin Dm_mit_) [37], [39]–[40]. *In vitro*, lamin Dm polymers can be solubilized by cdc2, PKC (protein kinase C) and PKA kinase (protein kinase A) as well [11].

Early, syncytial embryo specific *in vivo* phosphorylation on soluble lamin Dm was demonstrated using mass spectrometry. The identified phosphopeptide suggested location of one of the phosphorylation sites on S^45^ residue within the sequence of typical, lamin N-terminal cdc2 kinase motif [41]. In older (6–18 h) embryos identified phosphopeptides suggested S^19^, S^25^, S^595^ and T^435^ (candidate cdc2/PKC/PKA site) as the late embryo *in vivo* phosphosites [41]–[42]. Our study on embryos and Kc cells demonstrated that *in vivo* more phosphorylation sites may be involved and that they may be distributed along the entire protein [37].

It is commonly known that one of the main functions of the phosphorylation of lamins is to facilitate nuclear envelope breakdown, which probably occurs due to effects on the ability of lamins to polymerize. But we do not know whether, and if so which, other functions and interactions of lamins, such as chromatin binding or lamin location during mitosis, are affected by mitotic phosphorylation. Moreover, we do not know anything about the role of the interphase phosphorylation of lamins and if there is any difference in the function of phosphorylation between B and A type lamins. Besides, for better understanding of lamin functions it is important to know what is the exact effect of phosphorylation of a particular site. We know that lamins may be phosphorylated on the same site by different kinases and typically up to three phosphorylation sites are modified at the same molecule (lamin Dm) so different combinations of phosphorylated sites may result in different phenotypic effects. It is essential to know the effect of single sites before we start to elucidate the function of simultaneous phosphorylation on many sites, as it is in vivo. That is why we started the project of generation of “stably phosphorylated” (pseudophosphorylated) lamin Dm mutants. The idea was to use a simple model system to mimic single phosphorylation of a particular known *in vivo* site by its conversion into glutamic acid (e.g. S^25^E, S^45^E, S^595^E, T^435^E in lamin Dm). Thus, we were able to see the effect of single site modification with respect to major lamin properties (e.g. polymerization, chromatin binding, location and transport).

## Results

### Prediction of phosphorylation patterns on lamin Dm and lamin C

*Drosophila* lamins may be used as a good model for studying the effect of phosphorylation on both types of lamins. Because to date there are no reports about experimentally confirmed phosphosites on the fruit fly lamin C, we used Clustal W for detection of potential, evolutionary conservative sites located in this protein. The results showed that S^45^ in lamin Dm and S^37^ in lamin C lie within the very conservative, classical cdc2 phosphorylation motif present in all lamins. The tail domain of *Drosophila* lamins contains two typical regions of phosphorylation sites: one located immediately next to the coiled-coil domain and near the NLS sequence and the second at the end of the tail domain. The site containing T^435^ represents a potential cdc2 site but this is not a typical, conservative cdc2 site present in vertebrate lamins (Figure 1). Instead, T^435^ lies near or within a less conserved region responsible for interaction of lamin Dm with histones H2A and H2B *in vitro* (TRAT sequence) [34] and the NLS sequence. Lamin C has no cdc2 motif in this region, and the nearest possible cdc2 site is represented by S^405^ (S^405^PGR). Thus *Drosophila* lamins lack a typical, conservative C-terminal cdc2 motif characteristic of vertebrate lamins but possess a classical N-terminal cdc2 site.

Hence for the current study we decided to choose the following lamin proteins: wild-type lamin C and lamin C S^37^E mutant representing modification of the N-terminal cdc2 site as a control for A-type lamin. For lamin Dm study we used wild type protein, lamin Dm S^25^E, lamin Dm S^45^E, lamin Dm T^435^E and lamin Dm S^595^E. We also used lamin Dm R^64^H as an additional, control protein that is very well characterized *in vitro*, and unable to polymerize longitudinally [8]–[9], [11], [13], [43]. All the above mentioned proteins were bacterially expressed and affinity purified to high homogeneity (Figure S1).

### Modification of single phosphorylation sites affects solubility of lamins

In order to assess the secondary structure of bacterially expressed lamins, we analyzed their circular dichroism (CD) spectra (Figure S2). All wild type lamins and their mutants appeared to be well folded, containing domains with the helical coiled-coil conformation, and have the appropriate thermal denaturation curves. Lamin T^435^E was more resistant to thermal denaturation than wild type protein, while lamin Dm S^595^E was the least resistant. The calculated contents of secondary structures of analyzed proteins are shown in Table S1.

**Figure 2:**
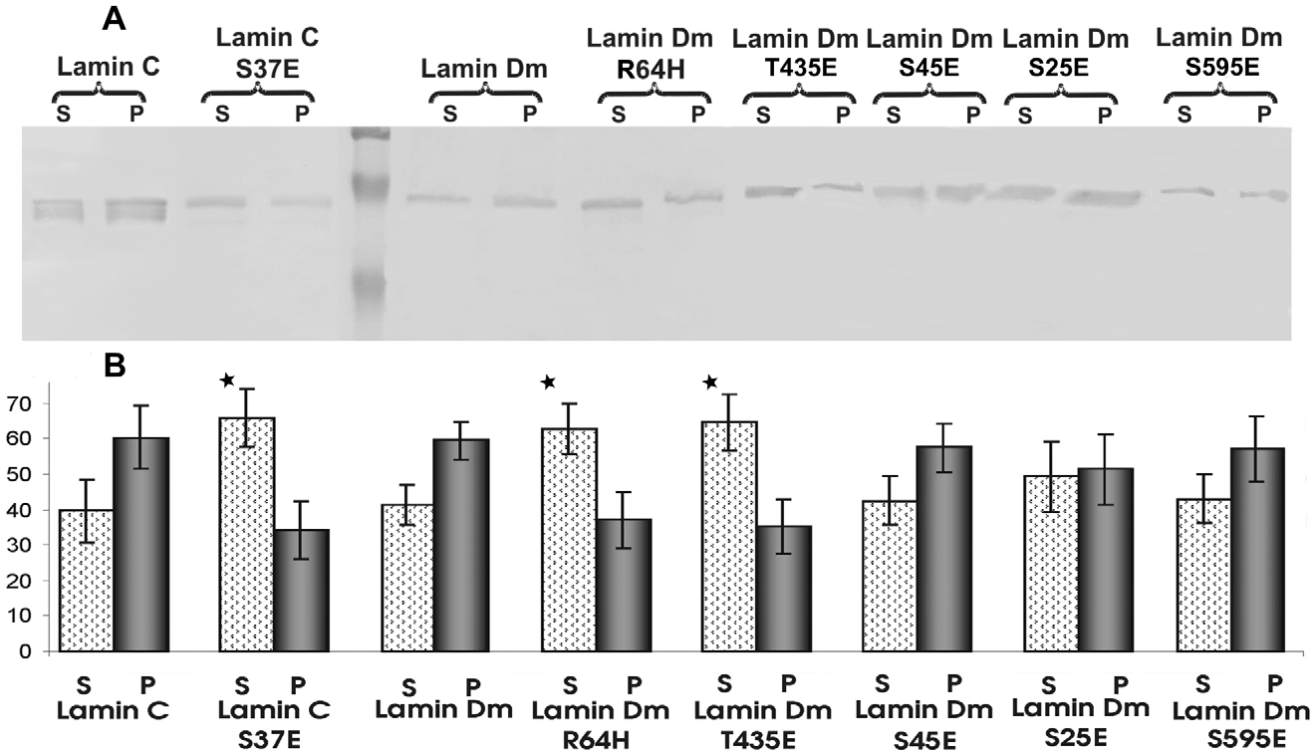
Phosphorylation of lamins changes their solubility *in vitro*. Panel A shows the typical result of sedimentation assay demonstrating polymerization ability of lamins. Proteins were allowed to polymerize for 60 min and centrifuged at 12 000×g for 20 min. Equal amounts of pellet and supernatant fractions were resolved onto 10% SDS PAGE followed by western blot with antibodies against *D. melanogaster* lamins. Panel B shows the diagram from the data obtained after quantification of the sedimentation assays. Data were obtained from three independent experiments.

The only well-documented function of specific *in vivo* phosphorylation of lamins is to affect lamin polymerization. We decided to confirm which particular lamin Dm phosphosites are responsible for modulation of polymerization using single pseudophosphorylation mutants. We examined the solubility of lamins in a sedimentation assay using western blotting for lamin detection in the pellet fraction (insoluble, presumably polymerized or in paracrystals) and in the supernatant fraction (soluble, presumably unpolymerized form) (Figure 2). As an additional control we also used lamin Dm mutant R^64^H, which was reported as a polymerization deficient mutant [8]–[9], [11], [13], [43]. Most lamin proteins demonstrated similar solubility. The exceptions were lamin Dm R^64^H, lamin Dm T^435^E and lamin C S^37^E. Lamin Dm mutant S^45^E representing the conserved N-terminal cdc2 phosphorylation site demonstrated polymerization similar to lamin Dm. Modification of the C-terminal cdc2 site on lamin Dm (T^435^) increased solubility of the protein. This indicates that phosphorylation of the C-terminal alone, but not the N-terminal conserved cdc2 site on lamin Dm may be sufficient for solubilization of lamin Dm *in vitro*.

### Only lamin C S^37^E mutant mimicking phosphorylation on the N-terminal cdc2 site does not bind to chromatin *in vitro*

**Figure 3:**
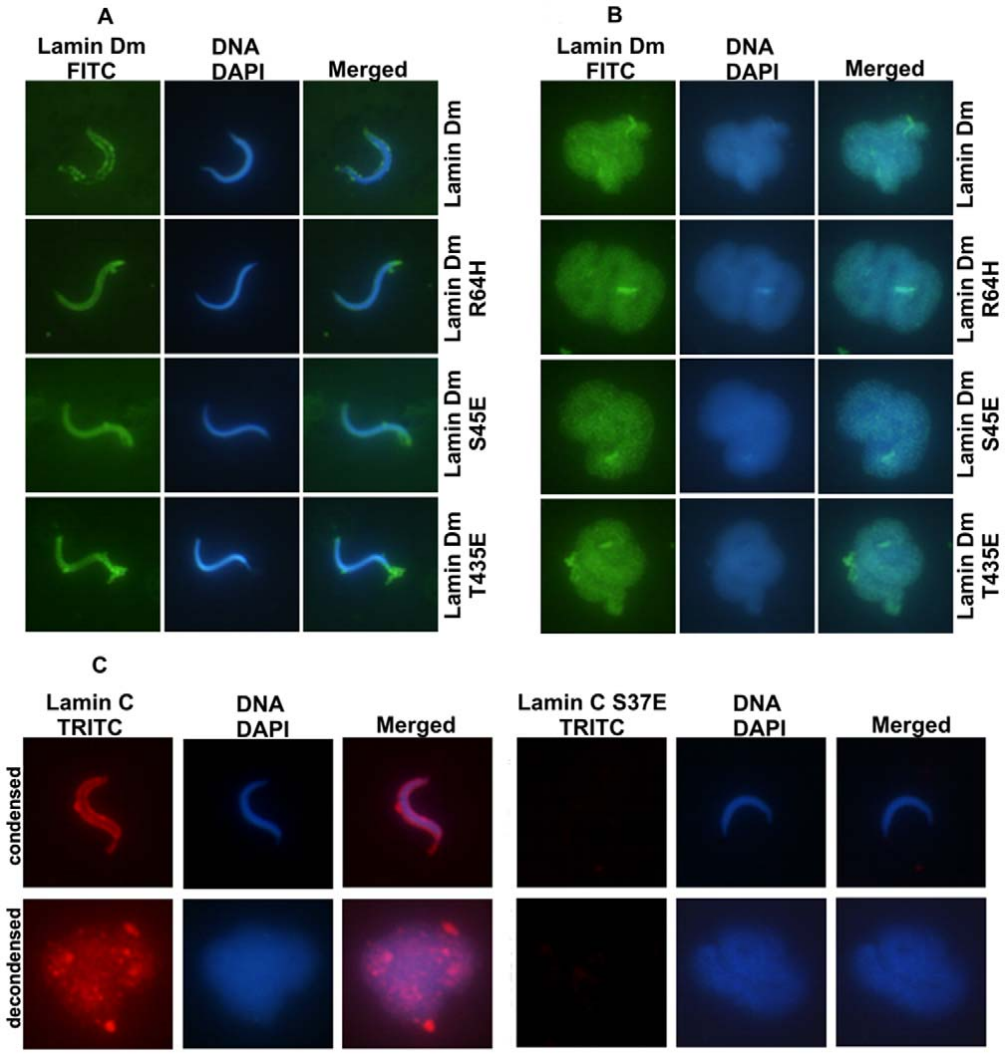
All bacterially expressed lamin proteins except lamin C mutant S^37^E bind *Xenopus* chromatin *in vitro*. Isolated *Xenopus laevis* sperm chromatin in condensed and decondensed form was used to assess the binding properties of lamin proteins and N-terminal fragment of *Xenopus* LAP2 protein. Lamin proteins were incubated with condensed and decondensed sperm chromatin for 15 min at room temperature. Preparations were then fixed in PBS buffer containing 1 mM EGS followed by spin down through a glycerol cushion onto a glass coverslip and processed for immunofluorescence microscopy analysis.

Another expected feature of lamin phosphorylation *in vivo* should be the loss of interaction between lamins, chromatin and nuclear envelope components. To test this assumption for *Drosophila* lamins, we used isolated *Xenopus* sperm chromatin as a target for lamin binding *in vitro*. Only lamin C S^37^E mutant failed to bind to chromatin *in vitro* (Figure 3), while the other tested pseudophosphorylated lamins and control proteins were able to bind to both condensed and decondensed chromatin, with similar efficiency, as revealed by IF studies. It is worth mentioning that lamin Dm R^64^H and T^435^E mutants, which had the same solubility as lamin C S^37^E, did bind to both forms of chromatin. Similar data were obtained on isolated HeLa interphase chromatin, permeabilized, interphase HeLa nuclei and chromatin isolated from late *Drosophila* embryos (not shown) as well as when chromatin was decondensed using interphase extracts from unfertilized *Xenopus* eggs. All lamin Dm proteins were able to associate with *Xenopus* pronuclei (Figure S3) while lamin C S^37^E protein did not (Figure S4).

### Lamin Dm S^45^E and T^435^E show different distribution in transfected *Drosophila* S2 and human HeLa cells

In order to test the *in vivo* functions of lamin phosphorylation, we constructed wild type lamin Dm and pseudophosphorylated mutants as EGFP (enhanced green fluorescent protein) fusion proteins. As a control, we used human wild type lamin A, B and C also as EGFP fusion proteins (Figure S5).

**Figure 4:**
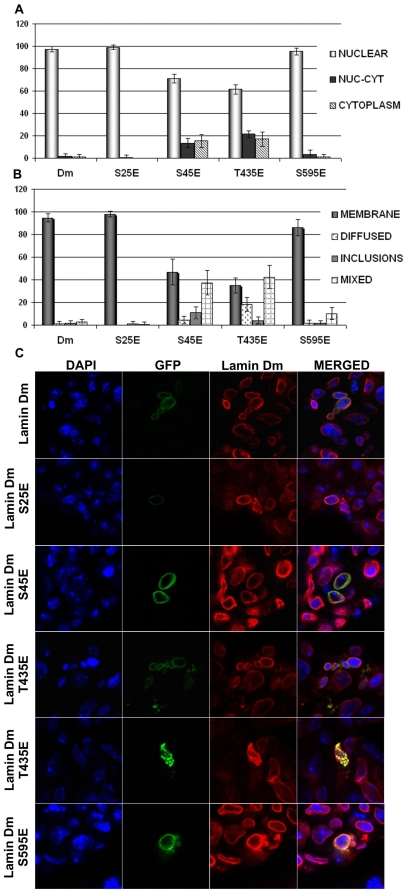
All lamin Dm mutants localize to nuclear lamina in transfected *Drosophila* S2 cells but lamin Dm S^45^E and T^435^E mutants show significantly different distribution. Localization of fusion GFP-lamin Dm and mutant proteins after 48 h (C) post-transfection into *Drosophila* S2 cells visualized under a confocal microscope and quantitative analyses of appearance of the particular phenotypes (A and B). Cells were stained for DNA with DAPI, for endogenous lamin Dm with mouse monoclonal antibodies ADL67. Exogenous lamin Dm proteins were visualized by eGFP fluorescence. Panel A demonstrates statistical analyses of distribution of lamin fusion proteins in nucleus only in nucleus and cytoplasm and in cytoplasm only. Panel B demonstrates statistical analyses of fusion proteins' localization to nuclear envelope and nuclear lamina (membrane), diffused phenotype, inclusion bodies phenotype and mixed phenotype respectively. Single confocal sections through the center of nuclei are shown. 200 cells were analyzed.

Overexpression of lamin Dm, lamin Dm S^25^E and S^595^E in S2 cells resulted in location of these proteins in the nucleus and in the nuclear lamina, where they colocalized with endogenous lamin Dm (Figure 4A, B, C). Lamin Dm mutants S^45^E and T^435^E showed similar location to each other but different from the rest of the mutants. Although lamin Dm S^45^E and T^435^E mutants generally localized to the nucleus, a significant amount of cells demonstrated nucleocytoplasmic and cytoplasmic locations (about 40%) of these proteins. This suggests that lamin Dm S^45^E and T^435^E mutants have impaired ability to be incorporated into the cell nucleus and the nuclear lamina compared to lamin Dm and other mutants. Since all lamin Dm mutants bind to chromatin *in vitro* and lamin Dm T^435^E does not polymerize while lamin Dm S^45^E polymerizes *in vitro*, this suggests that for lamin Dm location in the nuclear lamina *in vivo*, at least one property other than polymerization ability and chromatin binding ability is important as well. This also indicates that phosphorylation of S^45^ (N-terminal cdc2 site) alone is not sufficient for solubilization of lamin Dm *in vivo*, at least in S2 cells. Similarly, the phosphorylation of T^435^, which affects polymerization *in vitro*, has only a moderate effect on location of this mutant *in vivo*.

In order to test the generality of the lamin Dm interactions and due to the use of *Xenopus* sperm chromatin and HeLa chromatin in our *in vitro* studies, we overexpressed the same lamin Dm mutants in HeLa and *Xenopus* XTC cells. As a control, we used human wild type lamin A, B and C. Figure S5 demonstrates typical localization of wild-type human lamins after transfection of HeLa cells. Depending on the level of expression, human lamin-GFP proteins localize to the nucleus only, or the nucleus and surrounding space, forming nuclear envelope blebs and infrequently decorating ER structures.

**Figure 5:**
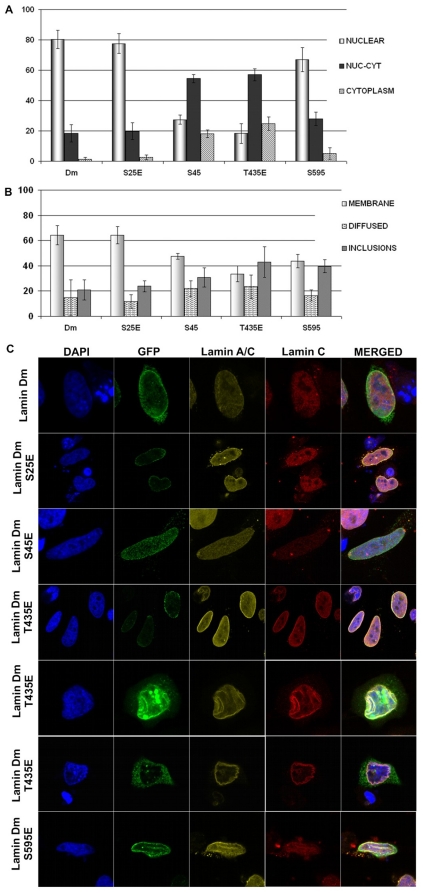
All lamin Dm mutants localize less efficiently to nuclear lamina in transfected HeLa cells but lamin Dm S^45^E, T^435^E and S^595^E mutants show significantly the lowest association with nuclear envelope. Localization of fusion GFP-lamin Dm and mutant proteins after 48 h (C) post-transfection into HeLa cells visualized under confocal microscope and quantitative analyses of appearance of the particular phenotypes (A and B). Exogenous lamin Dm proteins were visualized by eGFP fluorescence. Panel A demonstrates statistical analyses of distribution of lamin fusion proteins in nucleus only, in nucleus and cytoplasm, and in cytoplasm only. Panel B demonstrates statistical analyses of fusion proteins' localization to nuclear envelope and nuclear lamina (membrane), diffused phenotype, inclusion bodies phenotype and mixed phenotype respectively. Single confocal sections through the center of nuclei are shown. 200 cells were analyzed from at least 10 observation fields. Cells were stained for DNA with DAPI, for endogenous lamin A/C with mouse monoclonal antibodies Jol-2 and for lamin C alone with rabbit affinity purified antibodies. Staining with secondary antibodies was with goat anti-mouse secondary antibodies conjugated with TRITC and goat anti-rabbit secondary antibodies conjugated with Cy-5 respectively.

Wild type lamin Dm-GFP and mutants S^25^E and S^595^E demonstrated predominant nuclear location while S^45^E and T^435^E mutants were located both in the nucleus and cytoplasm. Over 20% of cells showed, beside nuclear, also cytoplasmic location of these mutants (Figure 5A, B, C). All lamin Dm proteins showed lower ability to be incorporated into nuclear lamina in HeLa cells when compared to *Drosophila* S2 cells but T^435^E mutant was the least effectively incorporated into the nuclear lamina. This mutant also showed the highest frequency of cytoplasmic location. One should note that mutant S^595^E, although it showed nuclear location, showed similarly decreased localization at the nuclear lamina as S^45^E and T^435^E mutants. These data support our hypothesis that factors other than polymerization ability and chromatin binding participate in affecting lamin Dm location *in vivo* both in *Drosophila* and in human cells. Surprisingly, S^45^ and T^435^ (also S^595^E) modification had a greater effect on location of lamin in HeLa cells than in *Drosophila* cells. This may indicate the additional role played by interacting protein partners of lamins in their nuclear lamina retention. In *Drosophila*, abnormal location is less frequent, probably due to the interactions, while in human cells lamin Dm may not interact or interact with lower affinity. Observed differences can also be connected with different physiological temperatures of human and insect cells lines, which can affect the protein properties (e.g. synthesis, folding, interactions). It should be pointed out that transfection of wild type human lamin A, B1, C as well as lamin Dm and mutants into HeLa cells similarly decreased the mitotic index of cells (below 0.5%) and induced apoptosis. The highest level of apoptosis (about 35%) was shown by lamin Dm T^435^E, the lowest by lamin A (about 10%). Preliminary analyses of cell cycle progression in connection with cytoplasmic/nuclear location of lamins do not show any specific correlation. There is, however, a correlation between the level of overexpression of exogenous protein and apoptosis. An approximately 50% decrease is observed in the number of cells highly overexpressing exogenous lamin between 24 h and 48 h. This effect is due to the increased rate of apoptosis in such cells and is characteristic for all lamins, both human and fly.

Detailed analyses of distribution of lamin Dm and endogenous lamins in HeLa cells demonstrated that most lamin Dm does not colocalize with endogenous lamins (A, B1 and C) at the nuclear envelope. Lamin Dm proteins localize at the nuclear envelope as small “domains” or form bigger fragments up to “budding” fragments of nuclear envelope (not shown).

### Lamin Dm T^435^E is not imported into the cell nucleus and shows higher dynamics *in vivo*

In order to test the hypothesis that the effect of lamin Dm pseudophosphorylation can be moderated or hidden by the effect of farnesylation of the C-terminal CAIM motif and nuclear or nuclear lamina retention may be the effect of nuclear envelope targeting by the farnesyl moiety, we prepared the same set of mutants (as EGFP fusion proteins) but with additional mutation in the C-terminus which prevents farnesylation.

**Figure 6:**
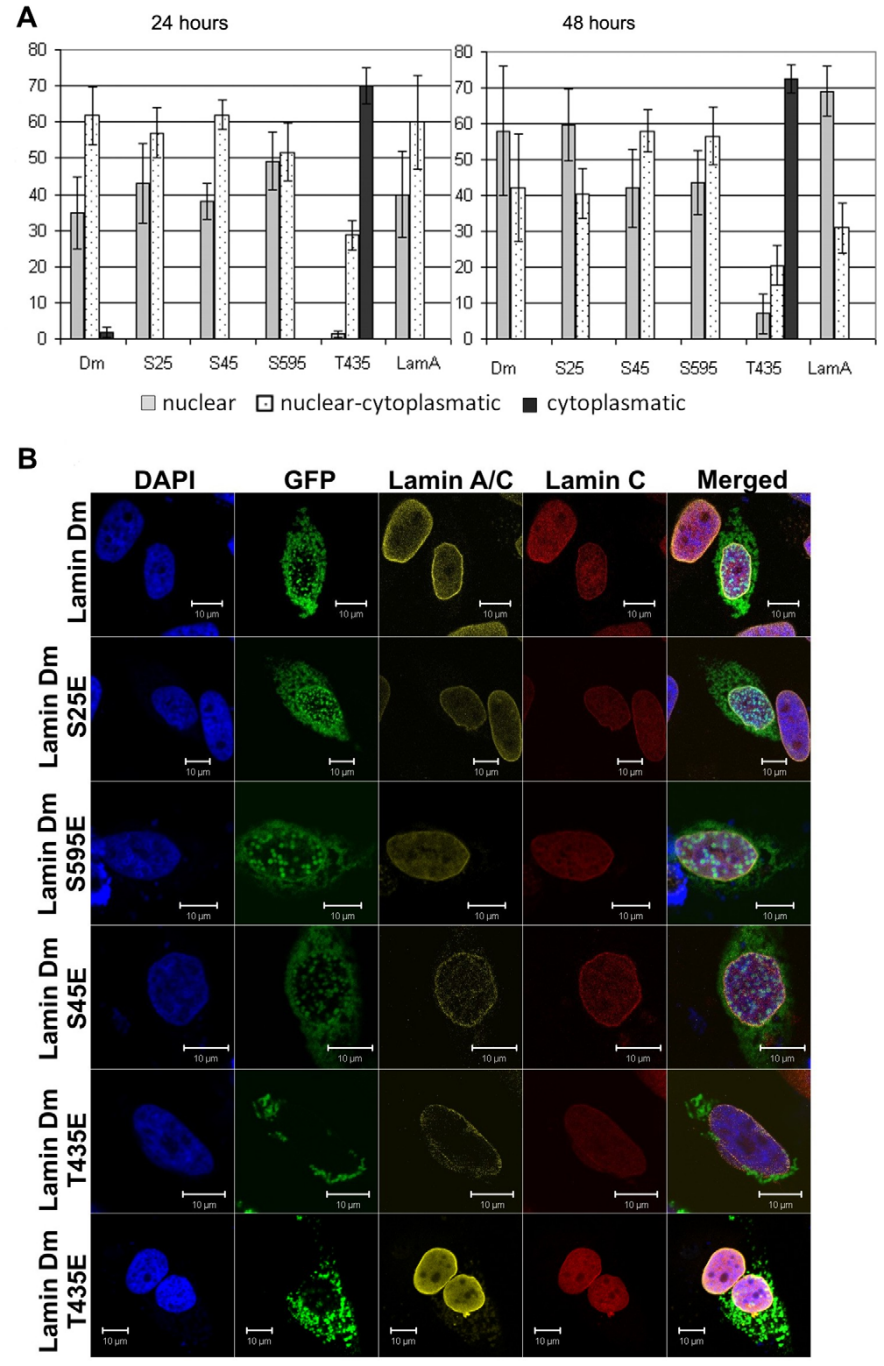
Farnesylation incompetent lamin Dm mutants do not localize efficiently to nuclear lamina and nuclear envelope. Localization of fusion GFP-lamin Dm and mutant proteins after 24 h (A) and 48 h (B) post-transfection into HeLa cells visualized under a confocal microscope and quantitative analyses of appearance of the particular phenotypes. Cells were stained for DNA with DAPI, for endogenous lamin A/C with mouse monoclonal antibodies Jol-2 and for lamin C alone with rabbit affinity purified antibodies. Staining with secondary antibodies was with goat anti-mouse secondary antibodies conjugated with TRITC and goat anti-rabbit secondary antibodies conjugated with Cy-5 respectively. Lamin Dm S^25^E and T^435^E were visualized by eGFP fluorescence. Single confocal Z-sections are shown through the center of nuclei. All typically observed phenotypes are shown for lamin Dm T^435^E mutant. Note the localization of lamin Dm T^435^E in the cytoplasm only.

Results of statistical analyses of transfected HeLa cells after 24 and 48 h are shown in Figure 6A. The amount of cells with nuclear or both nuclear and cytoplasmic location is similar for lamin Dm-GFP and lamin A-GFP transfection after 24 and 48 h and their nuclear location increases over time.

The most profound effect was observed for lamin Dm T^435^E mutant, which was predominantly cytoplasmic after 24 and 48 h post-transfection (Figure 6B). This indicates that lamin Dm T^435^E mutation affects nuclear import of lamin Dm and/or its retention in the nuclear lamina. This effect was undetected when the mechanism of nuclear transport and retention in the nuclear envelope through farnesylated C-terminus was active.

In order to gain insight into mobility of transfected fusion proteins and polymerization ability of lamins *in vivo*, we performed FRAP (fluorescence recovery after photobleaching) analysis. Experiments on transfected S2 cells and transgenic flies (embryos and salivary glands from larvae) expressing wild type lamin Dm-GFP demonstrated that lamin Dm in transgenic flies and all mutants in S2 cells have the same mobility. The mobility was similar to mobility of human lamin B-GFP transfected into HeLa cells. It is noteworthy that our previous experiments indicated that lamin Dm mutants transfected into S2 cells were predominantly nuclear and that farnesylation of lamin Dm facilitates its targeting to the nuclear envelope. This may affect mobility independently of polymerization ability of a particular lamin Dm mutant *in vivo*.

**Figure 7:**
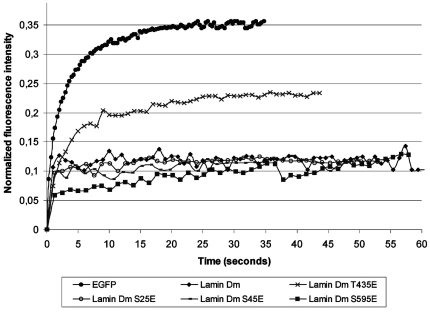
Graphical presentation of the data collected during FRAP experiments with farnesylation incompetent lamin Dm mutants in HeLa cells. Lamin Dm and all pseudophosphorylated mutants except lamin Dm T^435^E show the same low diffusion rate. Lack of recovery after photobleaching was observed for control human lamins (A, C and B1) (data not shown) as well as *D. melanogaster* wild type lamin Dm and the majority of lamin Dm mutants (S^25^E, S45E, S^595^E), excluding lamin Dm T^435^E. They showed no measurable recovery after several minutes. In contrast, lamin Dm mutant with threonine 435 substituted by glutamic acid to mimic stable, permanent phosphorylation displayed increased dynamics (D = 2.7 µm^2^/s; Mf = 20%). The presented fluorescence recovery curve shows that *Drosophila* lamins show similar mobility as human lamins in HeLa cells. Only specific mutation (T^435^E) can increase protein mobility, indicating lower polymerization ability *in vivo*. For control experiments we used HeLa cells expressing free EGFP. EGFP expression was seen to be evenly distributed within the nucleus and cytoplasm. Cytoplasmic fraction of EGFP shows a slower recovery (t1/2 = 2.05 seconds) versus that observed in the nucleus (t1/2 = 1.2 seconds).

Therefore we decided to measure the mobility of farnesylation incompetent lamin Dm mutants, using the FRAP method. We expressed lamins as fusion proteins with EGFP in HeLa cells. In FRAP experiments, fluorescent molecules were irreversibly photobleached in a defined area by a single, high-powered laser pulse. Next, the recovery of the fluorescent signal in the bleached regions was measured and expressed as the recovery. Figure 7 shows a graphical presentation of the collected data. All recombination proteins had a lower mobility than free EGFP. The wild type lamin Dm, and the majority of pseudophosphorylated mutants as well as human lamins showed similar mobility regardless of localization to the NE, nuclear interior structures or blebs of NE. What is worth noting, the lamin Dm T^435^E fusion protein showed higher mobility than other tested mutants or wild-type lamins and only about two times lower than nuclear EGFP alone.

## Discussion

In order to keep the experimental system as simple as possible we decided to analyze only single phosphorylation sites, although *in vivo* several sites may be phosphorylated on a single molecule [37] and they may act synergically [21]. For similar reasons we did not analyze the negative mutants (e.g. S or T into A). The potential phosphosites lay very close, so the mutation of a single phospho-acceptor may not prevent phosphorylation on the neighboring one – e.g. *in vivo* lamin Dm can be phosphorylated on S^25^ and S^595^ [41] but also on S^19^ and T^597^ [42]. Thus to have a “truly” unphosphorylated control for a single positive phosphorylation we would have had to mutate all serines and threonines within the entire region or protein itself. Since most of our studies are performed *in vitro* and no phosphorylation is possible, wild type lamins seem to be an adequate control. Besides, for sterical reasons, threonine should have been substituted by an amino acid with an identical side chain, which does not exist. Moreover, substitution of serine or threonine for alanine may affect the structure or interaction of such a mutant. A recent example might be the identical effect of mutations T^435^D and T^435^A in a 30 kDa lamin Dm fragment interacting *in vitro* with histones [34].

### Polymerization of *Drosophila* lamins

Since no data concerning mapping of *in vivo* phosphorylation sites on lamin C have been published, in our study we used only lamin C S^37^E mutant, representing the putative N-terminal cdc2 site. Based on the structure and the sequence of lamins, S^37^ seems to be the equivalent of the identified *in vivo* S^22^ in mammalian lamin A, S^28^ from goldfish lamin A [44] and S^45^ in lamin Dm. The lamin C region containing the C-terminal cdc2 site did not show such a high level of similarity to other lamins, as the N-terminal region shows (Figure 1).

In vertebrates, mitotic lamin phosphorylation is localized at two cdc2 (cdk1) sites flanking the rod domain [20]–[21], [45]. *D. melanogaster* lamins have only an N-terminal typical cdc2 kinase site (Figure 1). The potential C-terminal cdc2 sites are located differently in lamin Dm and lamin C as well as the vertebrate lamins.

*In vitro* lamin Dm can be either depolymerized or its polymerization inhibited by cdc2 kinase, PKC or PKA [11]. Also single point mutants of lamin Dm (R^64^C, R^64^H, I^396^V) showed decreased polymerization ability [8], [11], [13], [43], [46]–[47], demonstrating that single amino acid substitution itself can affect *D. melanogaster* lamin polymerization *in vitro*.

The lack of the effect of modification of the N-terminal cdc2 kinase site at lamin Dm on the polymerization is supported by the similar data obtained for vertebrate B-type lamins where C-terminal *in vivo* phosphorylation sites were detected for mitotic lamin B [48]–[49]. This confirms that lamin Dm is a typical B-type lamin in this respect.

Similarly, A-type lamins from vertebrates were reported to be phosphorylated on N-terminal or both N- and C-terminal sites during mitosis or while solubilized *in vitro* [21], [50]. This in turn is reflected by our data demonstrating that S^37^ pseudophosphorylation is sufficient for inhibition of polymerization of lamin C *in vitro*. Our discovery that phosphorylation of lamin C on the N terminal cdc 2 site is sufficient (to the same extent as lamin Dm phosphorylation on the C terminal cdc 2 site) for increasing solubility *in vitro* (Figure 2) may explain different behaviour of A and B-type lamins and may reflect the evolutionary adaptation to different functions of both types of lamins in the cell.

The presented FRAP data also indicated that pseudophosphorylation of T^435^ is sufficient for increasing solubility and mobility of lamin Dm *in vivo* (Figure 7). Preliminary experiments using fluorescence correlation spectroscopy (FCS) in an *in vitro* experimental setup with bacterially expressed lamins with Alexa 488 (data not shown) also support our thesis. The FCS data indicated that increased mobility of lamin Dm mutant T^435^E *in vivo* is the result of decreased polymerization since *in vitro* diffusion times for lamin Dm T^435^E and Dm R^64^H were similar (2.2 ms and 3.57 ms respectively; P<0.05) and ten times lower than for lamin Dm (32.15 ms; P<0.05). Similar differences in diffusion were obtained in FRAP experiments.

The last report on the *in vivo* phosphorylation on lamin Dm in fly embryos suggested S^45^ (N-terminal cdc2 site) and T^432^ or T^435^ (C-terminal cdc2 site) as phosphosites during embryogenesis with molar stoichiometry of about 0.75 and 0.2 mol phosphate/mol protein respectively [41]. Phosphorylated S^45^ was identified in the soluble lamin Dm fraction in early (0–5 h old) embryos. The C-terminal cdc2 site (T^432^/^435^) was identified in the late embryos in the lamin Dm fraction, which was insoluble. These results seem, at the first look, to be in contradiction to what should be expected about the function of B-type lamin phosphorylation at the cdc2 site (e.g. S^405^ is a predominant “mitotic” phosphorylation site on human soluble lamin B1 *in vivo*) [48]. Our data also indicate that single phosphorylation of the C-terminal cdc 2 site (T^435^), but not the N-terminal site (S^45^) is sufficient for lamin Dm solubilization. There may be several plausible explanations for such differences. Typically, lamin Dm in somatic cells is phosphorylated on two to three sites [37], [41] so identification of only a single site at the submolar ratio does indicate that other phosphosites had to be present on lamin Dm protein (and had not been identified), which might affect lamin polymerization. Note that not only phosphorylation of a particular mitotic site but also dephosphorylation of interphase sites may be essential for lamin solubilization [24]. An interesting feature of the syncytial embryo is that mitosis is very fast, synchronous and propagates in waves from one pole of the developing embryo. An additional explanation is that early embryos contain vast quantities of maternally provided lamin Dm as supplies for fast syncytial mitotic divisions. Such lamin Dm is gradually used up and recruited into newly created syncytial nuclei in the developing embryo. Besides, mitosis in syncytial embryos is “semi-closed”, which means that the nuclear envelope is depolymerized only at spindle poles at metaphase and disassembles almost completely only briefly at anaphase, forming a structure called the “nuclear spindle envelope” [51]. So lamin Dm modification in *Drosophila* early embryos on S^45^ may serve either as a storage form of maternally provided lamin Dm or as a necessary component of semi-closed mitosis in syncytial *Drosophila* embryos (with other unidentified phosphosites) or both. Moreover, early embryo soluble lamin Dm named Dm_mit_ was found to be copurified with a 90 kDa protein [52] or 90 kDa and 84 kDa proteins [42]. Interaction with these proteins may keep the lamin Dm fraction soluble. Additional support for soluble lamin inducible by interactions with protein comes from the reports about interaction of lamin A phosphorylated on S^404^ interacting with nuclear 14-3-3 protein [53]. This thesis is supported by an *in vitro* experiment in which interphase insoluble lamin Dm can be made soluble by addition of anti-lamin Dm IgGs [52]. Thus, lamin Dm S^45^ is probably not the only early embryo (syncytial) site and definitively not the only site in somatic cells because mutant S^45^E has the same properties as wild type lamin Dm. Instead, T^435^ residue seems to be the best available candidate, since mutant T^435^E is soluble.

Among all tested lamins, only lamin C S^37^E perfectly fits the ideal model of the effect of mitotic phosphorylation. This single site mutant was soluble, was not able to bind to chromatin or the nuclear envelope, and was unable to associate with the reforming cell nucleus in *in vitro* nuclear assembly. This indicates a much stronger effect of the N-terminal cdc2 site phosphorylation than in vertebrate A-type lamins, where both cdc2 sites have to be modified to observe the full effect [21].

Perhaps, the reason why S^45^ mutant and T^435^ mutant show only partial mislocalization in S2 cells is that farnesylation, by providing the mechanism for nuclear lamina retention as well as the possibility for ER lateral transport into the nucleus, “hides” the effect of phosphorylation on lamin location (Figure 4 versus Figure 5).

### Lamins and nuclear import

Transient transfection studies on HeLa cells with farnesylation incompetent lamin mutants demonstrated that all lamin Dm proteins, except T^435^E mutant, localized to the nuclear envelope and the cell nucleus (Figures 5, 6). The most probable explanation for T^435^E mutant location in cytoplasm is the inhibition of the nuclear import of this protein. This suggestion is supported by a previous report on chicken lamin B2 indicating that phosphorylation on C-terminal PKC sites (S^410^, S^411^) in response to phorbol ester treatment significantly reduced the import of lamin B2 into the nucleus [27].

### Chromatin binding

Lamins bind chromatin components *in vivo* directly or indirectly. Lamin Dm interacts directly with DNA *in vivo* during interphase but not during mitosis [39]. *Drosophila* lamin C does not interact *in vivo* with DNA at all. *In vitro* invertebrate and vertebrate lamins bind scaffold associated or matrix attachment regions of DNA (SAR/MAR DNA) [54]–[55] and centromeric regions [56]–[57]. Lamins bind *in vitro* to chromatin or nucleosomes [58]–[63]. In the case of mammalian lamins A/C, the DNA binding domain was localized in the C-terminal domain [64], similar to *D. melanogaster* lamin Dm, which binds to chromatin through histones H2A and H2B [43]. A recent report demonstrated that the C terminal fragment (aa: 425–473) of lamin Dm protein binds *in vitro* to chromatin through histones H2A and H2B [34]. This peptide, containing a C-terminal cdc2 site and NLS, did bind to mitotic chromatin, while substitution of T^432^D, T^435^D but also T^432^A and T^435^A inhibited binding. The authors suggested the conservative sequence T^432^RAT is essential for binding. Deletion of the NLS sequence also decreased binding to chromosomes similarly to human lamin A binding to DNA [64]. In our experiments with full length *Drosophila* lamins, only lamin C mutant S^37^E did not bind to any chromatin. All other proteins did bind to chromatin from *Xenopus* (Figure 3; Figures S3, S4), HeLa cells and *Drosophila* embryos (not shown), which suggested that other regions of lamins may also be involved in interactions with chromatin. This binding ability is not simply dependent on polymerization since lamin Dm R^64^H and T^435^E did bind to chromatin. Lamins interact with chromatin in at least two modes, through nucleic acids and through histones, and each way of binding requires different lamin fragments for interaction. Thus phosphorylation of the C-terminal cdc2 site on lamin Dm, while possibly affecting interactions through histones, may not affect interactions with nucleic acids or other protein(s) associated with nucleic acids.

The lamin Dm sequence flanking from the C-end the C-terminal cdc2 site is the site which is necessary for binding to the chromatin through histones [34]. The TRAT (SRAT/S) motif is only present in lamin Dm and other B-type lamins, being absent from lamin C (GRVT) and A-type lamins from humans (GRAS) and *Xenopus* (ARTI) (Figure 1). Surprisingly, a similar motif to lamin Dm (RNS**T^432^RAT**PS) is present in the N-terminal fragment of lamin C (RV**S^13^RAS**TSTP). Phosphorylation of S^37^ on lamin C may affect a hairpin-like structure of the head domain of lamin C and “hide” this potential histone binding motif. The best example of such a sterical change may be the report that *in vitro* phosphorylation of S^25^ on lamin Dm inhibits binding of monoclonal antibody ADL84 to its epitope RPPS^25^AGP. But also single point mutations in lamin Dm protein distant to this epitope or on the rod domain (e.g. A^40^V, K^146^M) abolish antibody binding [8]–[13]. This suggests that S^37^ phosphorylation on lamin C may also induce conformational change in the head domain of lamin C, which in turn prevents binding to chromatin through histones.

Experimental data indicate that in vertebrates, lamins A/C reassociate with the decondensing chromatin and reforming nuclear envelope later than B-type lamins [65]–[66]. Our data demonstrate that lamin Dm and lamin C properties such as polymerization, chromatin binding, mobility and nuclear import may be regulated by phosphorylation. Moreover, phosphorylation regulates the mentioned properties of lamin Dm and lamin C in different ways. We suggest that the different effect of pseudophosphorylation on B-type and A-type lamin in flies observed by us may, at least partially, be responsible for the unique properties of both types of lamins during interphase and mitosis.

## Materials and Methods

### Protein sequences used for Clustal W analysis

Lamin Dm (AAF52262); Lamin C (NP_523742); Lamin-1 (CeLam-1) (Ce-lamin) (Q21443); *Xenopus laevis* Lamin B1 (AAC31543); Lamin B2 (AAC31544); Lamin-L (Lamin-B3) (P10999); Lamin-A (P11048). *Gallus gallus* (chicken) lamin B2 (NP 990616); *Homo sapiens* (human): Lamin B1 (AAA36162).

### Constructs

The expression vector pET23a xLAP2 1–165 for the N-terminal 165 aa fragment of *Xenopus laevis* LAP2 (xLAP2) protein was a kind gift of Prof. Kathrin Wilson (Baltimore, USA) [67]. The cDNA sequence of lamin Dm point mutant lamin Dm R^64^H in expression vector pET20b(+) was a kind gift of Prof. Yosef Gruenbaum (Jerusalem, Israel) [13].

### Cloning and mutagenesis

The full length *Drosophila melanogaster* lamin C coding sequence from previously cloned pET17xblamC vector [13] was subcloned into pET29b vector (Novagen) using PCR. Lamin Dm cDNA was subcloned into pET29b (Novagen) vector from pET3a vector [68]. Mutations were introduced by using the Stratagene QuikChange XL mutagenesis kit, according to the manufacturer's instructions. The mutagenic primers were: LamDmS25E: 5′-ccggccgcca**gag**gcgggtccgcagc-3′; 5-gctgcggacccgc**ctc**tggcggccgg-3′; LamDmS45E: 5′-ctccagccccctc**gag**cccacccggcactcg-3′; 5′-cgagtgccgggtggg**ctc**gagggggctggag-3′; LamDmT435E: 5′ctccacgcgagcc**gag**ccatcgcgtcgcac3′; 5′gtgcgacgcgatgg**ctc**ggctcgcgtggag3′; LamDmS595E: 5′ctgagtcgtcgtcgc**gag**gtgaccgccgtggacgg3′; 5′ccgtccacggcggtcac**ctc**gcgacgacgactcag3′; LamCS37-E: 5′cctcacccacc**gag**cccacgcgcaccagc3′; 5′gctggtgcgcgtggg**ctc**ggtgggtgagg3′. Sequences of the mutants were confirmed by DNA sequencing.

### Protein expression and purification

For protein overexpression in bacteria, the *E. coli* BL21DE3(pLysS) (Stratagene) strain was transfected with derived expression vectors. Protein expression was induced with 1 mM IPTG for 6 h. Bacteria were harvested by centrifugation, washed with PBS and suspended in PBS buffer containing 0.1% Tween 20. Bacterial cells were lysed by sonication, extracted on ice for 10 min followed by centrifugation at 12000 g for 15 min. Resultant supernatant was collected for analysis. The obtained pellet was extracted successively with PBS buffer containing 1% Triton X-100, 3 M urea and 6 M urea. His-tagged lamin fusion proteins were purified on Ni-NTA-agarose columns (Qiagen).

### Lamin polymerization

Proteins were incubated in PBS buffer at room temperature for 60 min at the concentration of 100 µg/ml. After incubation, samples were centrifuged at 12 000 g for 20 minutes. Pellet fractions were solubilized for SDS PAGE. Supernatant fractions were TCA precipitated (10% final concentration) for 10 min on ice and proteins collected by centrifugation (16 000 g; 20 min; 4°C). Resultant pellets were washed twice with ice cold acetone and solubilized for SDS PAGE. Pellets and supernatants from the sedimentation assays were analyzed in immunoblotting. Immunoblots were digitalized and analyzed in gel documentation equipment with a CCD camera and using Bio-Profil V99 software (Vilber Lourmat). Quantitative data were obtained from 3 independent experiments and were expressed as the ratio of the amount of a particular protein in the pellet or supernatant fraction compared to the total amount of protein in both fractions.

### Isolation of *Xenopus* egg extract and demembranated sperm chromatin

Egg collection and extract preparation were according to Salpingidou et al. [69]. For sperm chromatin isolation male frogs were pre-stimulated with two portions of 150 iU of HCG (Human Chorionic Gonadotropin; Sigma) 48 and 24 hours before sperm isolation.

### *Xenopus* sperm chromatin binding assay

Isolated and demembranated sperm chromatin (DSP) from *Xenopus* was used in the condensed form and in the decondensed form prepared by incubation of sperm chromatin with poly L-glutamic acid in Pfaller buffer [70] for 20 min at room temperature. Sperm chromatin decondensation was performed as follows: chromatin of about 10^5^ sperm was incubated in Pfaller buffer with 0.5 µg/µl poly L-glutamic acid for 20 min at room temperature in a total volume of 40 µl. For sperm chromatin binding assays the bacterially expressed proteins (0.25 µg–1 µg) were added to the condensed or decondensed chromatin (about 10^5^) in extraction buffer in a total volume of 50 µl. The reaction proceeded for 15 min at room temperature. Chromatins were recovered on glass coverslips and processed for immunofluorescence analyses according to Drummond et al. [71]. Images were taken using an inverted Olympus IX70 fluorescence microscope.

### Work with animals

The Laboratory of Nuclear Proteins has all necessary documents, licenses and the Local Ethical Committee license for work with *Xenopus laevis*. The Local Ethical Committee License for experiments with *Xenopus* is permanent and was issued on 02.07.2003 (Number of application: 216/03; Number of license: 75/03). The Polish National Ethical Committee (KKE) issued a license to the Faculty of Biotechnology, University of Wrocław on behalf of the Laboratory of Nuclear Proteins for animal maintenance and performing experiments on animals (License Number KKE/36/2007/pl; Issued 14.03.2007). The Laboratory of Nuclear Proteins has certification from the Polish Ministry of Science and Higher Education for cultivation of animals and performing research (Decision No. 9/2007; Identification number 110).

### *Xenopus in vitro* sperm pronuclei assembly

*Xenopus* sperm pronuclei were reconstituted according to Salpingidou et al. [69]. In tests with bacterially expressed lamins added to the reaction, typically between 0.25 µg and 1 µg of protein was added. Assembly reactions were visualized with an Olympus IX70 fluorescence microscope or Olympus FV500 laser confocal microscope.

### Lamin Dm staining in tissue cultured cell

HeLa cells or *Xenopus* XTC cells were grown on 12 mm cover slips (in a 50 mm culture dish plate) up to 80% confluency as described previously [72]. S2 cells were cultured as described previously [37]–[38]. Cells were fixed with 4% formaldehyde, permeabilized with 0.5% Triton X-100 in PBS, and after washing with PBS were incubated for 20 minutes at room temperature with 25 µl of PBS containing 0.5 µg of lamin. After incubation, cells were washed, fixed and processed for immunofluorescence. Primary antibodies were used for human lamin C (affinity purified anti-lamin C specific fragment), *Drosophila* lamin Dm (ADL 67) and lamin C (ALC 28). The HeLa cell line was obtained from Ludwik Hirszfeld Institute of Immunology and Experimental Therapy of the Polish Academy of Sciences in Wrocław. The XTC cell line was obtained from the Integrative Cell Biology Laboratory, School of Biological and Biomedical Sciences, University of Durham, UK.

### Nuclei isolation

Human cells or *Xenopus* XTC cells were trypsinized, centrifuged at 118× g for 5 min in 50 ml Falcon tubes and resuspended in NIB buffer nucleus isolation buffer (10 mM Tris HCl pH 7.6; 10 mM NaCl; 3 mM MgCl_2_; 0.05% Triton X-10; with protease inhibitors) 1 ml/10^6^ cells. After 15 minutes on ice, cells were homogenized and centrifuged through 30% sucrose in NIB in Eppendorf tubes (2000× g, 10 min, 4°C). Cell nuclei were suspended in NIB buffer. Isolation of cell nuclei from *Drosophila* embryos and S2 cells was described previously [37], [39].

### Lamin binding to isolated cell nuclei or chromatin

Between 1 and 0.25 µg of each protein was added to the 10^5^ of cell nuclei or equal amounts of chromatin in 20 µl of PBS and incubated for 20 min at room temperature. Fixation was in 100 µl of 1% EGS in PBS for 30 min at 37°C. Samples were layered over 30% glycerol in PBS-Tween (0.05%) and centrifuged onto 22 mm coverslips (2000×g, 10 min, 4°C), fixed with formaldehyde and processed for immunofluorescence.

### Antibodies

Donkey anti-rabbit-FITC conjugated and donkey anti-mouse-TRITC conjugated (Jackson ImmunoResearch) were used at a dilution of 1∶50. ADL67 mouse monoclonal anti-lamin Dm was used at a dilution of 1∶2. LC 28 mouse monoclonal antibodies against lamin C were used at a dilution of 1∶3. R389 rabbit serum against lamin C was used at a dilution of 1∶50 for IF. Affinity purified antibodies for bacterially expressed human lamin C were used at 1∶20 dilution. Affinity purified IgGs from rabbit serum against lamin Dm were used at a dilution of 1∶50 [73].

### CD spectra measurement

CD (circular dichroism) spectra were collected with a J-175 spectropolarimeter (Jasco) using 2 nm bandwidth and response time of 1 s. Sampling was every 1 nm. Each curve represents the medium value for 3 scans. All experiments were carried out using cuvettes of 0.1 cm path length. Ellipticity was expressed as mean residue ellipticity [θ] in deg cm^2^ dmol^−1^ using the following equation: [θ] = [θ_obs_/(10×lxc)]/n, where θ_obs_ is the observed ellipticity in degrees, l is the optical path length of the cell in cm, c is the protein molar concentration, and n is the number of residues in the protein. Measurements were performed in phosphate buffer (20 mM sodium phosphate buffer with 0.6 M urea, pH 8.0).

### Transfection and analyses

HeLa cells (human epithelial carcinoma cell line) were transfected by plasmids encoding lamins by using Metafectene reagent (Biontex Laboratories GmbH, Germany) according to the manufacturer's instructions. S2 cells (*Drosophila* Schneider 2 cells, Invitrogen) were transfected by electroporation with CLB-Transfection Device (Lonza), program CELL5, using approx. 10^7^ cells and 10 µg of plasmid DNA. Transfection efficiency was about 30–50% in the case of HeLa cells and up to 10% in S2 cells. Based on calculations performed on transfected HeLa, S2 and HEK293 cells (90% efficiency of transfection) followed by western blot and IF analyses, it is possible to estimate that most of the transfected HeLa cells expressed exogenous lamins at a comparable amount to endogenous lamins. It was similar in S2 cells.

For analysis of data on lamin location we counted cells with location of transfected fusion lamin proteins in nucleus only, in nucleus and cytoplasm, and in cytoplasm. For nuclear location we assumed that at least 75% of the label was in the cell nucleus. For mixed, nuclear and cytoplasmic location we assumed that the label in nucleus and cytoplasm was lower than 75%. For cytoplasmic location we assumed that at least 75% of the fusion protein label was in the cytoplasm. The data for location of lamin fusion proteins in nuclear membrane, inclusions, diffused through nucleus or cytoplasm, or with mixed phenotype were based on the following assumptions:

For counting cells exclusively in the nuclear membrane and inclusion categories, at least 65% of the label had to be in these locations respectively. As diffused phenotype cells were counted cells with predominantly diffused or dispersed label but also “mixed” phenotype – cells with the label in the nuclear envelope and/or inclusions but without obvious domination of either phenotype. The rationale for assigning such phenotypes as diffused was that a significant fraction of the label in such cells is frequently also “dispersed”.

### FRAP analysis

FRAP analyses were performed on transfected HeLa, XTC and S2 tissue cultured cells. HeLa cells (in MEM alpha supplemented with Glutamax) were previously adapted to grow at room temperature without carbon dioxide. XTC cells and S2 cells normally grow at room temperature without carbon dioxide. Cells were cultured on glass coverslips for 24 h and 48 h after transfection and analyzed under a 40× (water immersion) objective in culture medium under a Zeiss LSM 510 Meta microscope according to the manufacturer's instructions. For each mutant FRAP analyses were performed on lamin proteins located in the nuclear interior, nuclear envelope and cytoplasm (if available). Mobility analyses were performed many times for each compartment. For calculations, at least three measurements, of the best quality from each compartment were selected. We used FRAP analysis software (Zeiss LSM510 software - ZEN 2008) for experiment setup and data collection (prebleach imaging, laser pulse, recovery imaging, collection of data, etc.) and calculation of values for the mobile fraction (Mf measured in %) and time required for 50% fluorescence plateau recovery of the bleach zone (t1/2 measured in seconds), using normalized fluorescence intensity. The diffusion coefficient [D measured in µm^2^/s] was determined from the equation D = ω^2^/4t1/2, where ω is the radius of circular ROI and t1/2 is diffusion half time. FRAP experiments were performed on cells transfected with wild type human lamin A, B1 and C, wild type lamin Dm and pseudophosphorylation mutants fused to EGFP protein. Data demonstrated in Figure 7 were collected with lamin Dm farnesylation incompetent mutants since lamin Dm and all pseudophosphorylation mutants with wild type farnesylation sequence showed the same mobility and the same percentage of mobile fraction.

## Supporting Information

Figure S1 **Overexpression and purification of lamin Dm, lamin C and mutant proteins.** 12% SDS-polyacrylamide gel electrophoresis of fractions from the purification of wild type lamin Dm and Lamin Dm S^45^E (Panel A and B respectively) together with lamin C and mutant lamin C S^37^E (Panel C and D respectively) and all purified lamins (Panel E). The lamin proteins were overexpressed in *E. coli* BL21DE3(pLysS) bacterial cells. Bacteria were lysed and proteins were solubilized from inclusion bodies in buffer containing 6 M urea and purified by metal affinity chromatography on Ni-NTA-agarose columns. Please note that relative electrophoretic mobility of particular protein may vary from gel to gel and lane to lane due to the different proteins concentration, buffer composition, position etc. Panel E illustrates the quality of purified lamin proteins. M – Prestained Protein Molecular Weight Marker (Fermentas), BSA (bovine serum albumin) – 2.5 µg, S4 – proteins extracted in buffer containing 6 M urea, U – unbound fractions, E – elution fractions.
(TIF)

Figure S2 **Circular dichroism (CD) spectra of the purified wild type lamin C and lamin Dm, and their mutants.** Curves representing different temperatures are marked by different colours. Initial curves on both panels have two peaks, one at 208 nm and the second at 220 nm, demonstrating typical shape for native proteins containing a large proportion of α-helical structure. The denaturation was found to be largely reversible (R - renatured CD spectra).
(TIF)

Figure S3 **All lamin Dm proteins bind to chromatin and nuclear envelope in** ***in vitro*** **nuclear assembly system from** ***Xenopus*** **.** *In vitro Xenopus* sperm pronuclei assembly reaction was used to assess the ability of lamin Dm mutants to bind to assembling chromatin and nuclear envelope structures. Assembly reaction was carried out for 40 min in the presence of bacterially expressed proteins.
(TIF)

Figure S4 **Mutant lamin C S^37^E protein does not bind to** ***in vitro*** **assembled** ***Xenopus*** **sperm pronuclei.** *In vitro Xenopus* pronuclei assembly reaction was used to assess the ability of bacterially expressed lamin C and lamin C S^37^E mutant to bind to assembling chromatin and nuclear envelope structures. The control experiment was without addition of any exogenous protein. Assembly reaction was carried out for 30 min in the presence or absence of bacterially expressed proteins.
(TIF)

Figure S5 **Localization of fusion EGFP: lamin A, lamin B and lamin C 48 hours post-transfection into HeLa cells visualized under confocal microscope in order to visualise differences in phenotype depending on fusion protein expression level.** Cells were stained for DNA with DAPI and for lamin C with rabbit affinity purified antibodies. Staining with secondary antibodies was with goat anti-mouse secondary antibodies conjugated with TRITC and goat anti-rabbit secondary antibodies conjugated with Cy-5 respectively. Particular lamin fusion proteins were visualized by EGFP fluorescence. Single confocal Z-sections are shown through the center of nuclei. Only the most typical phenotypes are shown.
(TIF)

Table S1 **The content of distinct secondary structure types present in analyzed lamins and residue molar ellipticity.** The content of distinct secondary structures in lamin proteins was calculated using CDFIT software. Molar ellipticity values were measured at 20°C in PBS with 0.6 M urea.
(DOC)
