# Supplementary figures and images for: The Different Function of Single Phosphorylation Sites of Drosophila melanogaster Lamin Dm and Lamin C
Source: PLoS One. 2012 Feb 29;7(2):e32649. doi: 10.1371/journal.pone.0032649 (PMC3290585; doi:10.1371/journal.pone.0032649)

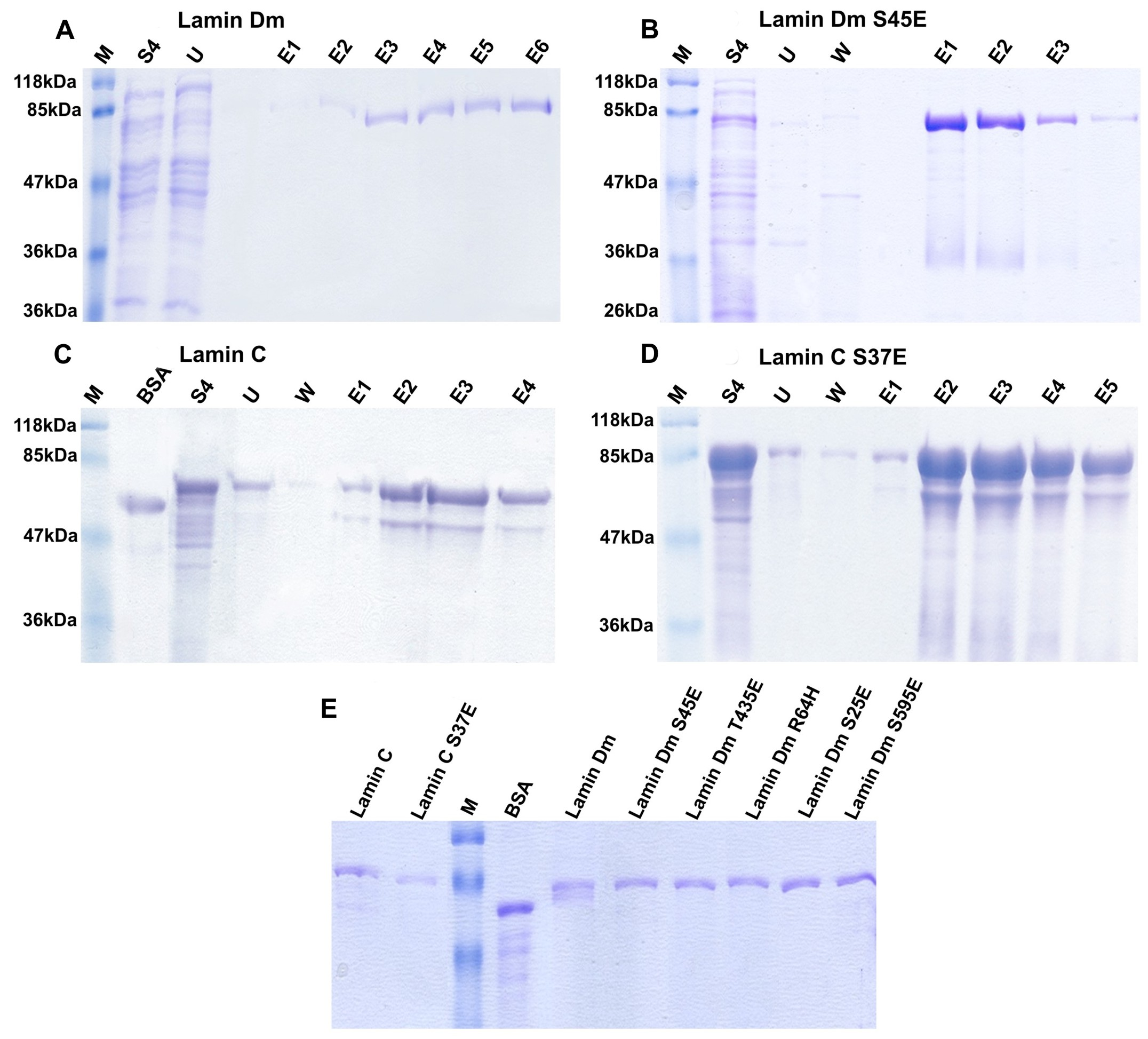

Supplement: Figure S1 — Overexpression and purification of lamin Dm, lamin C and mutant proteins. 12% SDS-polyacrylamide gel electrophoresis of fractions from the purification of wild type lamin Dm and Lamin Dm S45E (Panel A and B respectively) together with lamin C and mutant lamin C S37E (Panel C and D respectively) and all purified lamins (Panel E). The lamin proteins were overexpressed in E. coli BL21DE3(pLysS) bacterial cells. Bacteria were lysed and proteins were solubilized from inclusion bodies in buffer containing 6 M urea and purified by metal affinity chromatography on Ni-NTA-agarose columns. Please note that relative electrophoretic mobility of particular protein may vary from gel to gel and lane to lane due to the different proteins concentration, buffer composition, position etc. Panel E illustrates the quality of purified lamin proteins. M – Prestained Protein Molecular Weight Marker (Fermentas), BSA (bovine serum albumin) – 2.5 µg, S4 – proteins extracted in buffer containing 6 M urea, U – unbound fractions, E – elution fractions. (TIF) [file pone.0032649.s001.tif]

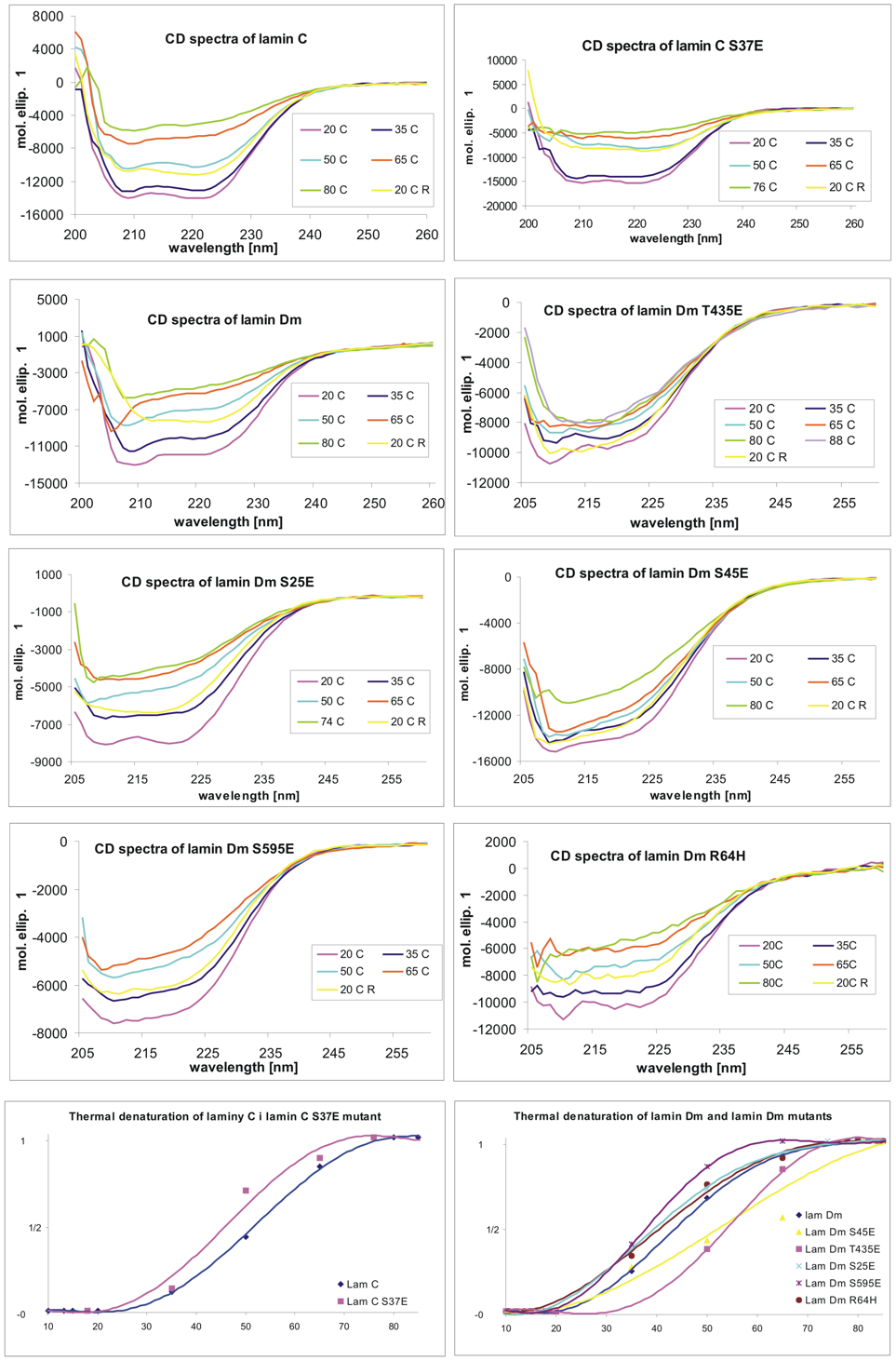

Supplement: Figure S2 — Circular dichroism (CD) spectra of the purified wild type lamin C and lamin Dm, and their mutants. Curves representing different temperatures are marked by different colours. Initial curves on both panels have two peaks, one at 208 nm and the second at 220 nm, demonstrating typical shape for native proteins containing a large proportion of α-helical structure. The denaturation was found to be largely reversible (R - renatured CD spectra). (TIF) [file pone.0032649.s002.tif]

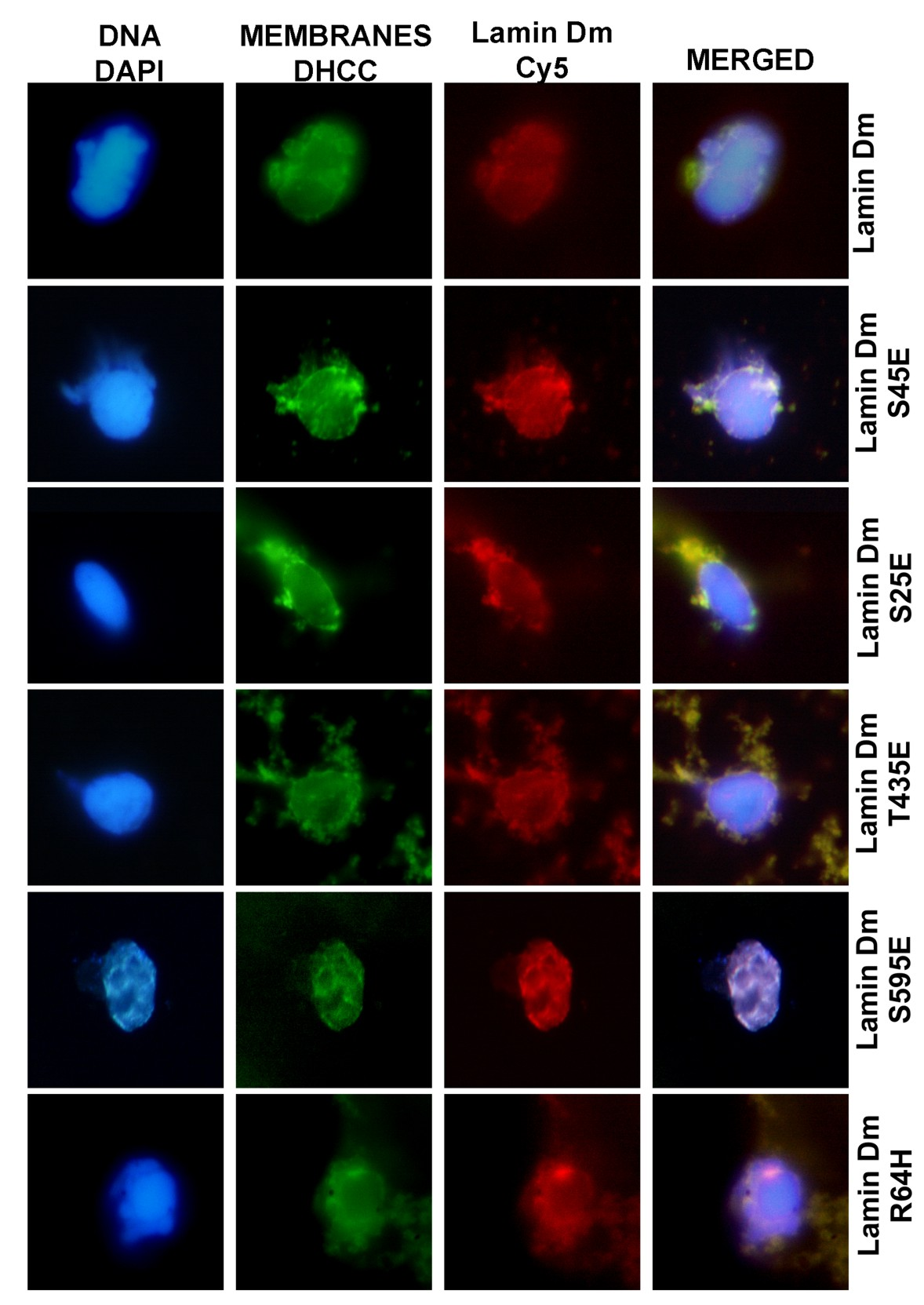

Supplement: Figure S3 — All lamin Dm proteins bind to chromatin and nuclear envelope in in vitro nuclear assembly system from Xenopus . In vitro Xenopus sperm pronuclei assembly reaction was used to assess the ability of lamin Dm mutants to bind to assembling chromatin and nuclear envelope structures. Assembly reaction was carried out for 40 min in the presence of bacterially expressed proteins. (TIF) [file pone.0032649.s003.tif]

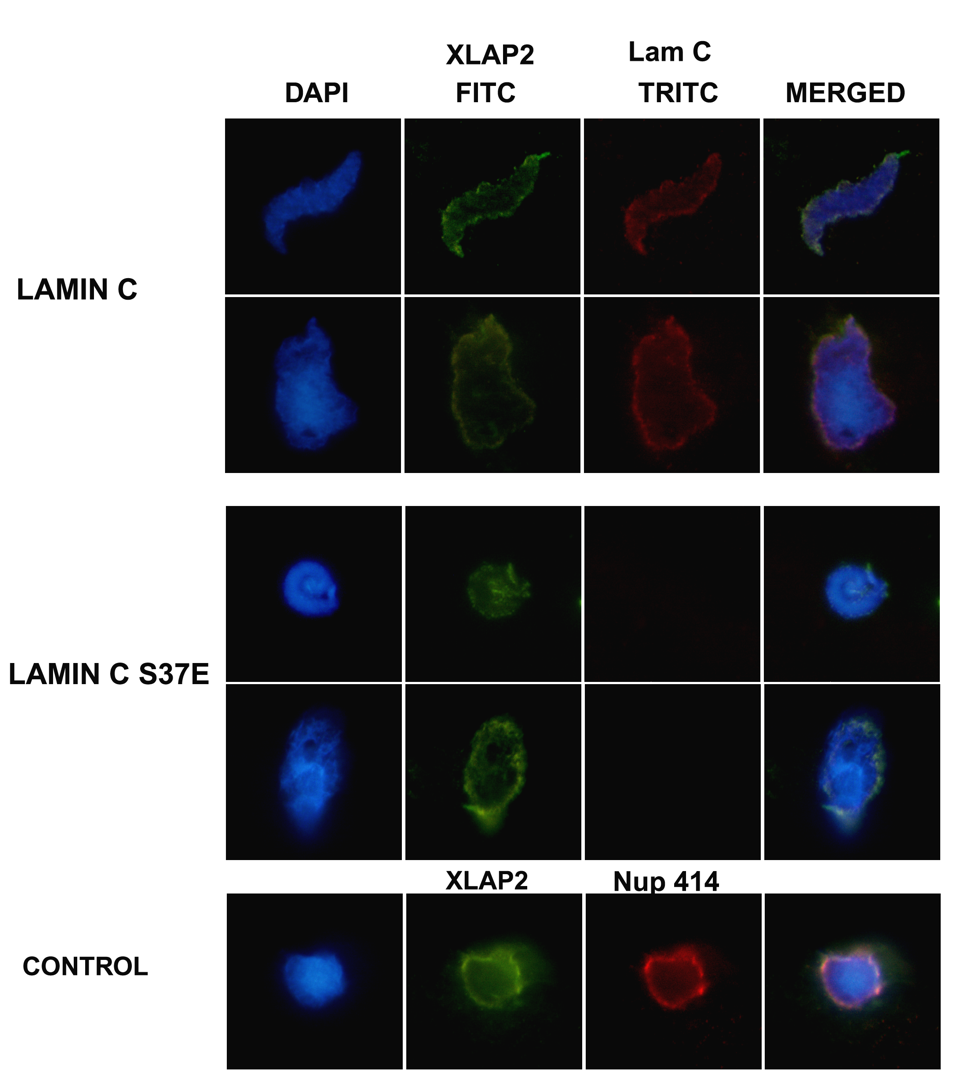

Supplement: Figure S4 — Mutant lamin C S37E protein does not bind to in vitro assembled Xenopus sperm pronuclei. In vitro Xenopus pronuclei assembly reaction was used to assess the ability of bacterially expressed lamin C and lamin C S37E mutant to bind to assembling chromatin and nuclear envelope structures. The control experiment was without addition of any exogenous protein. Assembly reaction was carried out for 30 min in the presence or absence of bacterially expressed proteins. (TIF) [file pone.0032649.s004.tif]

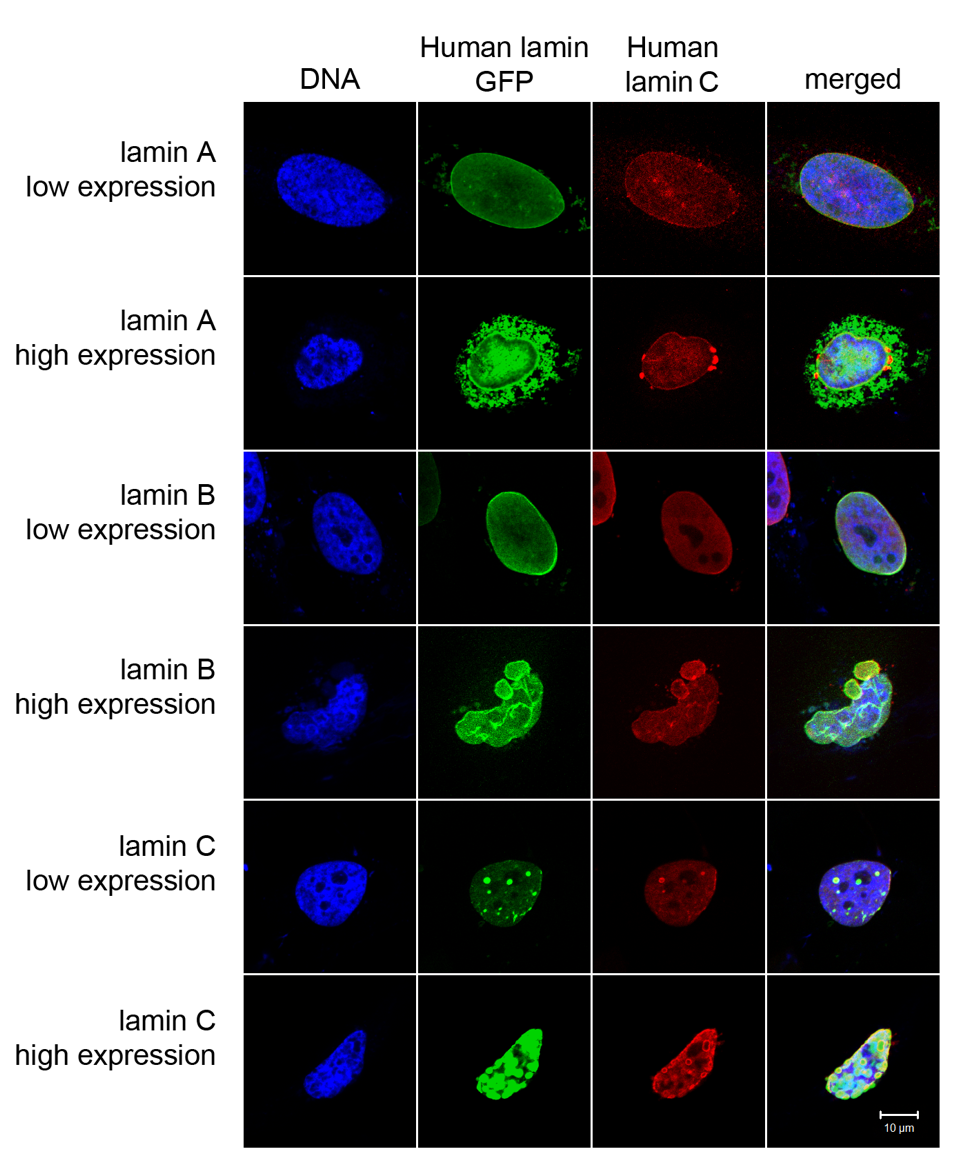

Supplement: Figure S5 — Localization of fusion EGFP: lamin A, lamin B and lamin C 48 hours post-transfection into HeLa cells visualized under confocal microscope in order to visualise differences in phenotype depending on fusion protein expression level. Cells were stained for DNA with DAPI and for lamin C with rabbit affinity purified antibodies. Staining with secondary antibodies was with goat anti-mouse secondary antibodies conjugated with TRITC and goat anti-rabbit secondary antibodies conjugated with Cy-5 respectively. Particular lamin fusion proteins were visualized by EGFP fluorescence. Single confocal Z-sections are shown through the center of nuclei. Only the most typical phenotypes are shown. (TIF) [file pone.0032649.s005.tif]
