# Supplementary material for: The Different Function of Single Phosphorylation Sites of Drosophila melanogaster Lamin Dm and Lamin C
Source: PLoS One. 2012 Feb 29;7(2):e32649. doi: 10.1371/journal.pone.0032649 (PMC3290585; doi:10.1371/journal.pone.0032649)
Supplement: Table S1 — The content of distinct secondary structure types present in analyzed lamins and residue molar ellipticity. The content of distinct secondary structures in lamin proteins was calculated using CDFIT software. Molar ellipticity values were measured at 20°C in PBS with 0.6 M urea. (DOC) [file pone.0032649.s006.doc]

| Protein | Total  α-helix content  [CDFIT] | Total  β-**sheet content** [CDFIT] | Total random coil content  [CDFIT] | Measured molar ellipticity at 222 nm at 20^o^C [degrees^.^cm^2.^dmol^-1^] |
| --- | --- | --- | --- | --- |
| lamin Dm | 28.85% | 25.72% | 45.42% | -11808 |
| lamin Dm S^25^E | 38.95% | 25.94% | 35.11% | -7961 |
| lamin Dm S^45^E | 25.08% | 33.77% | 41.16% | -13552.9 |
| lamin DmT^435^E | 20.46% | 32.51% | 47.03% | -9167.7 |
| lamin Dm S^595^E | 19.03% | 34.19% | 46.78% | -6943.7 |
| lamin C | 25.08% | 33.77% | 41.16% | -14023.9 |
| lamin C S^37^E | 38.80% | 25.49% | 35.71% | -15102.7 |

**Table S1. The content of distinct secondary structure types present in analysed lamins and residue molar ellipticity.** The content of distinct secondary structures in lamin proteins was calculated using CDFIT software. Molar ellipticity values were measured at 20^o^C in PBS with 0.6 M urea.
